# High content screening identifies monensin as an EMT-selective cytotoxic compound

**DOI:** 10.1038/s41598-018-38019-y

**Published:** 2019-02-04

**Authors:** Marion Vanneste, Qin Huang, Mengshi Li, Devon Moose, Lei Zhao, Mark A. Stamnes, Michael Schultz, Meng Wu, Michael D. Henry

**Affiliations:** 1 Department of Molecular Physiology and Biophysics, Roy J. and Lucille A. Carver College of Medicine, The University of Iowa, Iowa City, IA 52242 USA; 2 Department of Internal Medicine, Roy J. and Lucille A. Carver College of Medicine, The University of Iowa, Iowa City, IA 52242 USA; 3 Department of Radiation Oncology, Roy J. and Lucille A. Carver College of Medicine, The University of Iowa, Iowa City, IA 52242 USA; 4 Department of Biochemistry, Roy J. and Lucille A. Carver College of Medicine, The University of Iowa, Iowa City, IA 52242 USA; 5 Department of Pathology, Roy J. and Lucille A. Carver College of Medicine, The University of Iowa, Iowa City, IA 52242 USA; 6 Department of Urology, Roy J. and Lucille A. Carver College of Medicine, The University of Iowa, Iowa City, IA 52242 USA; 7 Human Toxicology, University of Iowa, Iowa City, IA 52242 USA; 8 University of Iowa High Throughput Screening Facility (UIHTS), University of Iowa, Iowa City, IA 52242 USA; 9 Division of Medicinal and Natural Products Chemistry, Department of Pharmaceutical Sciences and Experimental Therapeutics, College of Pharmacy, University of Iowa, Iowa City, IA 52242 USA; 10 Holden Comprehensive Cancer Center, University of Iowa, Iowa City, IA 52242 USA

## Abstract

Epithelial-to-mesenchymal transition (EMT) is implicated in cancer metastasis and drug resistance. Specifically targeting cancer cells in an EMT-like state may have therapeutic value. In this study, we developed a cell imaging-based high-content screening protocol to identify EMT-selective cytotoxic compounds. Among the 2,640 compounds tested, salinomycin and monensin, both monovalent cation ionophores, displayed a potent and selective cytotoxic effect against EMT-like cells. The mechanism of action of monensin was further evaluated. Monensin (10 nM) induced apoptosis, cell cycle arrest, and an increase in reactive oxygen species (ROS) production in TEM 4-18 cells. In addition, monensin rapidly induced swelling of Golgi apparatus and perturbed mitochondrial function. These are previously known effects of monensin, albeit occurring at much higher concentrations in the micromolar range. The cytotoxic effect of monensin was not blocked by inhibitors of ferroptosis. To explore the generality of our findings, we evaluated the toxicity of monensin in 24 human cancer cell lines and classified them as resistant or sensitive based on IC_50_ cutoff of 100 nM. Gene Set Enrichment Analysis identified EMT as the top enriched gene set in the sensitive group. Importantly, increased monensin sensitivity in EMT-like cells is associated with elevated uptake of ^3^H-monensin compared to resistant cells.

## Introduction

Metastasis is the major driver of mortality in cancer patients. It involves a series of events including localized stromal invasion, intravasation, transport through circulation, extravasation and colonization^1^. Considerable phenotypic changes in epithelial cells occur enabling these events. Epithelial to mesenchymal transition (EMT) is a mechanism in vertebrate development that is also responsible for dramatically, and reversibly, altering the phenotype of epithelial cells to enable morphogenesis and cell migration in the embryo^2^. EMT-like phenotypic changes, such as a loss of apico-basal polarity and epithelial markers and a gain of invasive motility and mesenchymal markers, are readily observed in cancer cell lines, cancer tissues and are experimentally demonstrated to confer metastatic behaviors *in vitro* and in animal models. However, the extent to which EMT is required for metastasis remains controversial^3,4^. EMT-like cells also exhibit resistance to a variety of therapeutic modalities^5^. Therefore, the process of EMT, and EMT-like cells themselves, present a potential target to thwart cancer progression and therapeutic resistance^6,7^.

The EMT-like phenotype can be targeted using various approaches. First, EMT-like changes can be blocked or prevented by targeting the inducing signals, reviewed in^8^. However, this approach is potentially challenging as such changes may occur early in disease progression, and because EMT-like characteristics can be induced by numerous stimuli. Reversing EMT by forcing mesenchymal to epithelial transition (MET) is another approach to revert cells to a less aggressive phenotype and to potentially sensitize cells to conventional therapies, reviewed in^8^. However, a caveat to this approach is that it may have the potential to facilitate metastatic outgrowth. Finally, selectively killing cells in an EMT-like state is expected to be useful in combination with conventional therapies to prevent the development of therapeutic resistance, reviewed in^8^.

Therapeutically targeting an EMT-like phenotype may be a new approach to treat metastatic disease, but it is not without many challenges^6,8^. In the past 10 years, several screens were conducted to identify compounds able to inhibit or reverse an EMT-like phenotype, reviewed in^8^. However, to our knowledge, no one has attempted a systematic high throughput screen for compounds with anti-EMT activity in a co-culture context. In this study, we developed a high content (cell imaging-based) high throughput screen (HTS) using two sub-populations of the PC-3 prostate cancer cell line (TEM 4-18 and PC-3E cells) in co-culture to identify compounds with a selective cytotoxic effect against an EMT phenotype. TEM 4-18 cells were isolated by virtue of their preferential ability to cross an endothelial monolayer in a model of metastatic extravasation^9^. These cells exhibit a ZEB1-dependent EMT-phenotype and are more aggressive in metastatic colonization *in vivo* than the parental PC-3 cell line. By screening two compound libraries comprising 2,640 compounds, we identified monensin as a potent EMT-cytotoxic compound. Our studies show that nanomolar levels of monensin (10 nM) effect rapid (6 h) and irreversible loss of clonogenic potential and concomitant disruption of the Golgi apparatus and perturbation of mitochondrial function in TEM-4-18 cells, but not PC-3E cells. These effects of monensin have been described previously in other studies, albeit at much higher (10-to1000-fold) concentrations. Monensin sensitivity is observed in cancer cells from diverse tissue origins that exhibit EMT-like phenotypes. Finally, we show that EMT-like cells exhibit greater uptake of monensin compared to cells with epithelial features, which may explain the high sensitivity of these cells to monensin.

## Results

### Strategy and optimization of high content screening

**Figure 1:**
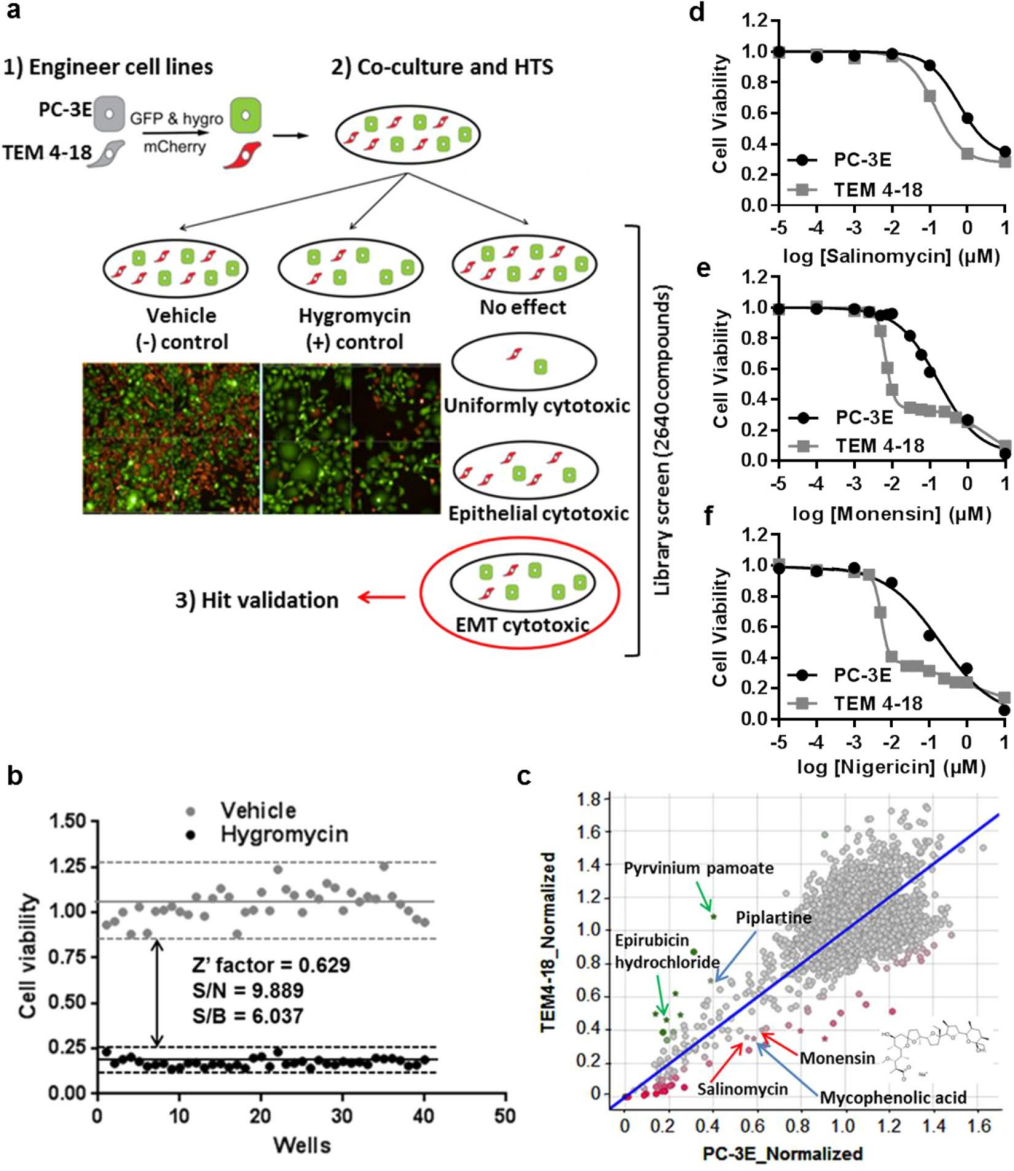
High content screen for EMT-selective compounds. (**a**) High content screen design. (**b**) Assay performance was assessed with a half-half plate using following parameters: the signal-to-noise (S/N) ratio = (µn − µp)/SDn, the signal-to-background (S/B) ratio = µn/µp, and the Z’ factor. (**c**) Results of the screen representing relative cell viability of TEM 4-18 mCherry (y axis) and PC-3E GFP (x axis) cells. Positions of selected compounds evaluated are indicated. (**d**–**f**) Cells cultured separately were treated for 72 h with serial dilution of salinomycin (n ≥ 5) (**d**), monensin (n ≥ 24) (**e**) or nigericin (n ≥ 5) (**f**). Relative cell viability was plotted against the logarithm of drug concentration. Data represent mean values ± SEM.

We developed and optimized a high content HTS for EMT-selective cytotoxic compounds using co-cultured PC-3E and TEM 4-18 cell lines (Fig. 1a). In order to discriminate PC-3E and TEM 4-18 cells easily, the two cells lines were labeled with GFP and mCherry respectively. Expression of GFP in PC-3E and mCherry in TEM 4-18 was confirmed by flow cytometry analysis (Supplementary Fig. S1). As a positive control for our assay, we also introduced a hygromycin-resistance marker into PC-3E GFP cells. Therefore, PC-3E GFP cells are hyg^R^ while TEM 4-18 mCherry cells are hyg^S^ (Fig. 1a). The differential expression of several EMT signature genes between engineered PC-3E and TEM 4-18 cell lines was confirmed by qRT-PCR (Supplementary Fig. S1). Cells were imaged with PerkinElmer Operetta system (Supplementary Fig. S1). Automated cell counting results were also validated by manual cell counting (Supplementary Fig. S1). The average Z’-factor was 0.63, S/N was 9.9 and S/B was 6.0, indicating that the quality of our cell-based assay was acceptable for high throughput analysis (Fig. 1b).

### High content screening identified salinomycin and monensin as potential EMT-selective cytotoxic compounds

**Figure 2:**
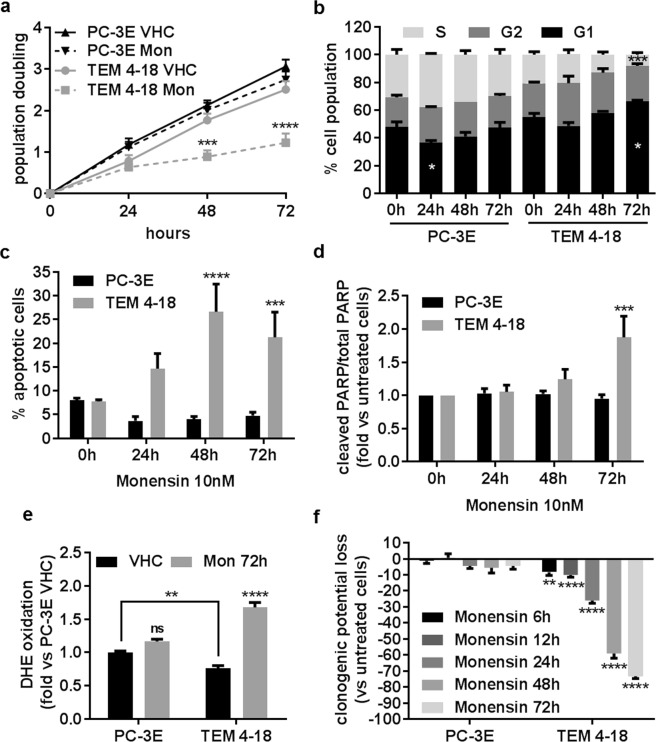
Monensin induces oxidative stress, apoptosis and a cell cycle arrest in TEM 4-18 cells. PC-3E and TEM 4-18 cells were treated with monensin (10 nM) for up to 72 h. Every 24 h, (**a**) the number of population doubling (n = 6), (**b**) cell cycle progression (PI incorporation) (n = 3), (**c**,**d**) apoptosis induction (C: Annexin V staining (n ≥ 3); D: PARP cleavage (n = 4)), (**e**) ROS formation (DHE probe oxidation) (n ≥ 7), and (**f**) clonogenic potential (n ≥ 12) were evaluated. Data represent mean values ± SEM.*p < 0.05, **p < 0.01, ***p < 0.005, ****p < 0.0001.

**Figure 3:**
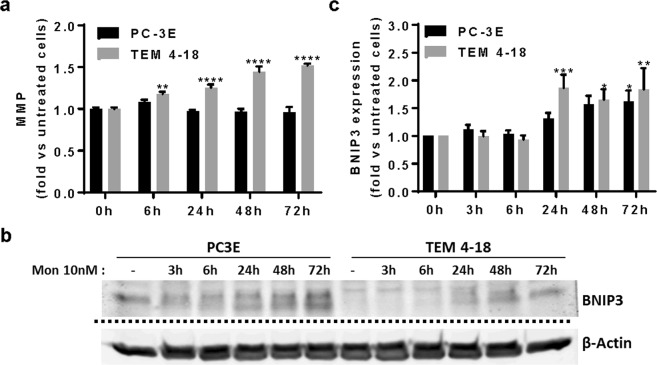
Monensin perturbs mitochondrial function and induces mitochondrial damage in TEM 4-18 cells. Cells were treated for 3 h to 72 h with monensin (10 nM). (**a**) Mitochondrial membrane potential (JC-1 staining) (n ≥ 8) and (**b**,**c**) expression of BNIP3 (Western blot) were evaluated (n ≥ 6). (**b**) shows a representative Western blot. The blot was cut based on migration of molecular weight markers and stained separately for BNIP3 and β-actin. The images shown are cropped from sections from the blots shown in Supplementary Fig. S6. Data represent mean values ± SEM.*p < 0.05, **p < 0.01, ***p < 0.005, ****p < 0.0001.

Following initial characterization of the assay, a screen was performed using the Spectrum library (2320 known biologically -active compounds) and the UI Legacy compound collection (320 compounds) at a final concentration of 1 µM for each compound. The majority of the compounds had either no effect on relative cell viability or slightly promoted the growth of one of the two cell lines (Fig. 1c). The different hits showing a large deviation from the negative controls can be categorized into three clusters: (1) Hits cytotoxic to both PC-3E and TEM 4-18 cells, at the bottom left corner of the Fig. 1c; (2) Hits preferentially cytotoxic to EMT-like TEM 4-18 cells, at the bottom right of the Fig. 1c; (3) Hits preferentially cytotoxic to PC-3E cells, at the top left of the Fig. 1c. With the aim of identifying EMT-selective cytotoxic compounds, we investigated compounds that had relatively greater cytotoxic effect against TEM 4-18 cells. Thus, 5 hits were selected based on the following criteria: (a) Green cells_normalized ≥0.55; (b) Red cells_normalized ≤0.41 and (c) Ratio R/G ≤ 0.65 (Fig. 1c). Interestingly, 6 compounds showed a selective cytotoxic effect on PC-3E GFP hygro cells and were selected by the criteria of: (a) Green cells_Normalized ≤0.41; (b) Red cells_normalized ≥0.46 and (c) Ratio R/G ≥ 1.80 (Fig. 1c). Only 9 of the 11 selected hits were commercially available and tested. Some additional compounds met these criteria but were intentionally disregarded because of their problematic chemical structure or interference with the assay (fluorescent compounds). As the screen was performed at a fixed concentration (1 µM), it is possible that we missed some hits that would display selectivity at a lower concentration. Therefore, it would be interesting to repeat the screen at a lower concentration for the compounds that displayed a high cytotoxicity against both cell populations (Fig. 1c). The results of the screen have been deposited in PubChem (https://pubchem.ncbi.nlm.nih.gov/bioassay/1345086). Dose response curves for 6 of the selected compounds are displayed in Supplementary Fig. S2. Among them, salinomycin and monensin, both monovalent carboxylic ionophores, showed significantly (p < 0.001) lower IC_50_ values for TEM 4-18 mCherry (18 nM and 76 nM respectively) compared to PC-3E GFP cells (2028 nM and 2636 nM respectively) (Supplementary Fig. S2). On the contrary, pyrvinium pamoate and epirubicin hydrochloride showed significantly higher IC_50_ for TEM 4-18 cells compared to PC-3E cells (Supplementary Fig. S2), the latter finding is consistent with EMT-like cells being resistant to conventional chemotherapeutic drugs. Mycophenolic acid and piplartine exhibited relatively similar IC_50_ on both cell types (Supplementary Fig. S2) and were considered as false positives. Finally, gentian violet showed selectivity against PC-3E cells while dactinomycin and daunorubicin displayed a high cytotoxicity on both cell lines (data not shown).

We then tested sensitivity to salinomycin (Fig. 1d) and monensin (Fig. 1e) on PC-3E and TEM 4-18 cells cultured separately using an independent cell viability assay. As both salinomycin and monensin belong to the family of the monovalent carboxylic ionophores, we evaluated nigericin, compound closely related but absent from the libraries tested to determine if differences in the ion selectivity profile of these compounds was related to their EMT-selective cytotoxicity (Fig. 1f). The highest potency and selectivity for the EMT-like cells was obtained with nigericin, (IC_50_ 194 nM for PC-3E vs 7 nM for TEM 4-18, 28-fold selectivity), followed closely by monensin (IC_50_ 186 nM for PC-3E vs 9 nM for TEM 4-18, 21-fold selectivity) with salinomycin being the least (IC_50_ 1564 nM for PC-3E vs 276 nM for TEM 4-18, 6-fold selectivity). Although all three compounds again demonstrated greater potency against the EMT-like TEM 4-18 cells, the IC_50_ values for salinomycin and monensin were somewhat different than those measured in the high content imaging assay (Supplementary Fig. S2), reflecting differences in the sensitivity to cell viability in the different assays or other variables.

### Monensin induces oxidative stress, apoptosis and cell cycle arrest in TEM 4-18 cells

To understand the mechanism(s) by which monensin selectively targets EMT-like cells, we evaluated the effect of the compound on cell cycle progression and apoptosis. For this purpose, we utilized a concentration of 10 nM which reduced TEM 4-18 cell viability by 50% at 72 h while having no effect on PC-3E cells. We first evaluated the effect of monensin on cell growth at 24 h, 48 h and 72 h. At this concentration, monensin did not affect the growth of TEM 4-18 cells for the first 24 h. However, cell growth was significantly decreased at 48 h and 72 h in TEM 4-18 cells while no effect was observed in PC-3E cells (Fig. 2a). These results were confirmed by examining cell cycle progression. Monensin induced a cell cycle arrest characterized by an accumulation of cells in G1 phase associated with a reduction of the cells in S phase after 72 h exposure, while no effect was observed on the PC-3E cells (Fig. 2b). In addition, the effects on cell growth at 48 h and 72 h were concomitant with induction of apoptosis in TEM 4-18 cells as determined by both annexinV staining and PARP cleavage (Fig. 2c,d). Intracellular reactive oxygen species (ROS) can regulate various intracellular signal transduction cascades and play an important role in induction of apoptosis^10^. Monensin (>1 µM) was previously described as potent inducer of oxidative stress^11^. Therefore, we explored the effect of monensin on ROS generation using oxidative fluorescent dye DHE to detect H_2_O_2_ and superoxide production. The fluorescence signal intensities attributable to H_2_O_2_ and superoxide production were higher in PC-3E cells than in TEM 4-18 cells at baseline (Fig. 2e). However, 72 h exposure to monensin increased markedly DHE oxidation in TEM 4-18 cells while having no effect on PC-3E cells (Fig. 2e). Taken together, these results indicated that monensin reduced viability of TEM 4-18 cells by inducing a cell cycle arrest and apoptosis associated with an increase of intracellular oxidative stress. Although cell growth of TEM 4-18 cells was not obviously affected by monensin at 24 h, we wondered whether shorter exposure to this compound might already be sufficient to reduce cell viability. Therefore, we investigated the effect of exposure to monensin shorter than 24 h on clonogenic potential. Our data indicated that monensin induced a loss of clonogenic potential of TEM 4-18 cells as early as 6 h exposure (Fig. 2f). Indeed, 6 h, 12 h, 24 h, 48 h and 72 h exposure to monensin decreased clonogenic potential of TEM 4-18 cells by 8%, 10%, 26%, 59% and 73% respectively while having no significant effect on PC-3E cells. These results indicate that irreversible deleterious effects of monensin on TEM 4-18 cells occurred within the first 24 h.

### Monensin induces mitochondrial damage in TEM 4-18 cells

It is well known that mitochondria play a key role in the formation of intracellular ROS and can trigger apoptosis^10^. There are several reports that monensin (>100 nM) can induce ultrastructural alteration of the mitochondria and cause the collapse of mitochondrial membrane potential which might be a critical step in monensin-induced apoptosis^12,13^. In order to test if monensin-induced apoptosis in TEM 4-18 was due to impairment of mitochondrial function, we measured the effect of monensin on the mitochondrial membrane potential of PC-3E and TEM 4-18 cells. Surprisingly, monensin (10 nM) did not cause a collapse, but on the contrary, induced an increase of the mitochondrial membrane potential. This increase was detected only in TEM 4-18 cells and was observed after 6 h exposure (Fig. 3a). To evaluate the deleterious effect of monensin on mitochondria in TEM 4-18 cells, we measured the expression of the mitochondria damage marker BNIP3. Interestingly, monensin induced the expression of BNIP3 after 24 h in both PC-3E and TEM 4-18, with BNIP3 levels being higher in PC-3E prior to monensin exposure (Fig. 3b,c). Although monensin affects mitochondria in both cell lines at 10 nM, these results suggest that PC-3E cells are not affected by this stress unlike TEM 4-18 cells.

### Golgi disruption is an early marker of monensin toxicity

**Figure 4:**
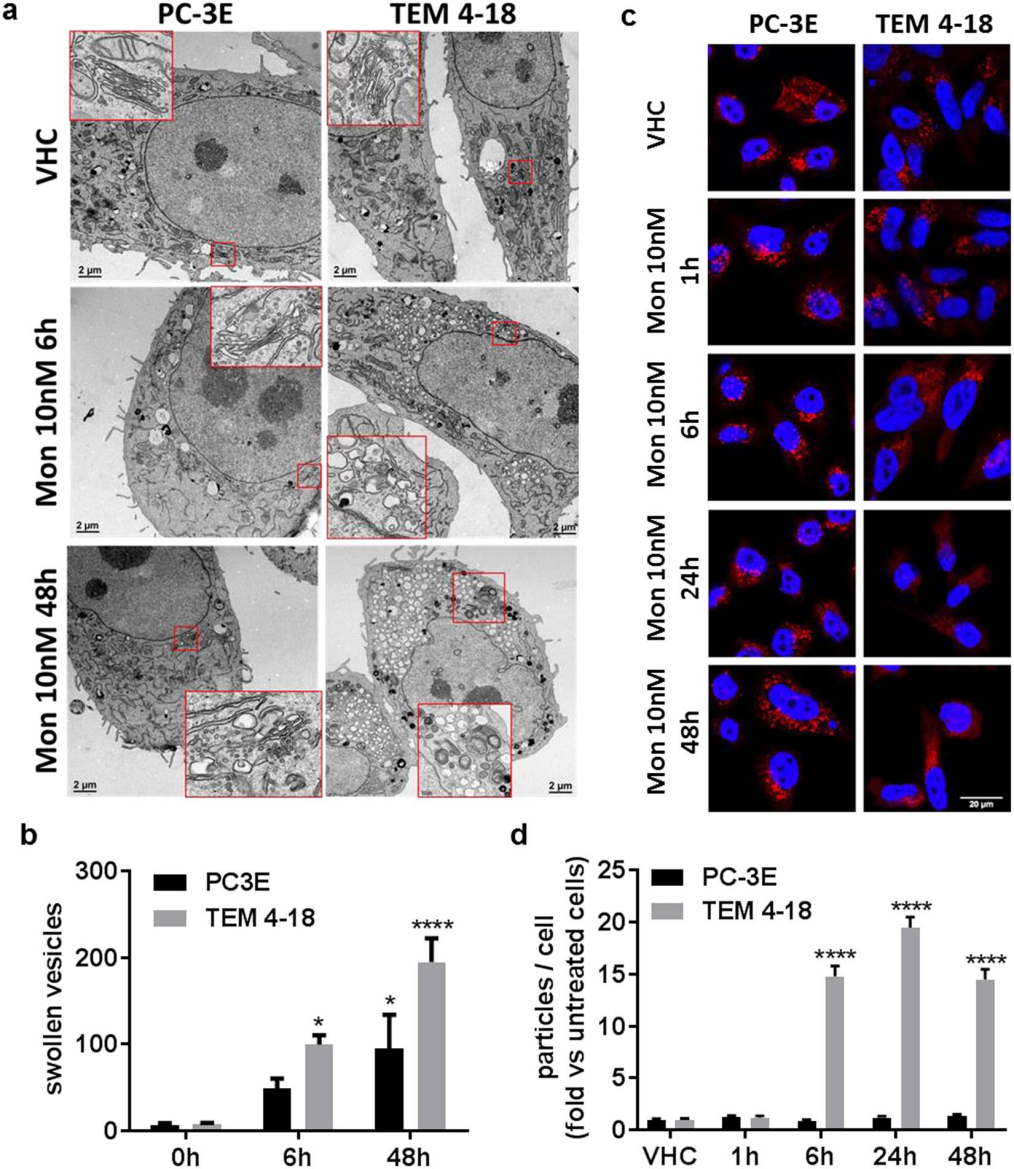
Golgi apparatus disruption is an early effect of monensin in TEM 4-18 cells. (**a**) Transmission electron microscopy was performed on cells treated with monensin (10 nM) for 6 h or 48 h. Scale bar: 2 µm. (**b**) Quantification of the number of swollen vesicles present in the cytoplasm (n ≥ 5). (**c**) Cells treated with monensin (10 nM) for 1 h to 48 h. Cells were stained for GM130 (red) and nucleus (blue). Scale bar: 20 µm. (**d**) GM130 signal was converted into binary signal and number of particles were counted using ImageJ and normalized to the number of particles in untreated cells (n ≥ 25). Data represent mean values ± SEM. *p < 0.05, **p < 0.01, ***p < 0.005, ****p < 0.0001.

Monensin (>100 nM) has also been extensively described as a Golgi disrupting agent, inducing a rapid swelling of Golgi apparatus and a perturbation of intracellular vesicular trafficking, reviewed in^14,15^. Therefore, we performed transmission electron microscopy experiments to assess the effect of monensin on Golgi apparatus morphology (Fig. 4a,b). In the vehicle treated cells, we observed abundant mitochondria, smooth endoplasmic reticulum as well as some degradation vesicles (lysosomes, autophagosomes, autophagolysosomes) in both PC-3E and TEM 4-18 cells. Normal Golgi structure was easily identifiable by the superposition of flattened cisternae in both cell lines (enlarged squares in a). Treatment for 6 h with 10 nM monensin did not affect the ultrastructure of smooth endoplasmic reticulum, mitochondria or degradation vesicles. However, treatment of TEM 4-18 cells resulted in the accumulation of dilated vesicles delimited by a single smooth membrane and mainly located in the perinuclear region. Normal Golgi stacks were no longer identifiable suggesting that the dilated vesicles could be derived from the Golgi apparatus as previously described^15^. On the other hand, these dilated vesicles were absent or less abundant in PC-3E cells in which the Golgi apparatus was still identifiable despite a noticeable enlargement of the cisternae. At 48 h, the number of dilated vesicles increased in TEM 4-18 cells as well as the number of degradation vesicles (enlarged square). While 100% of TEM 4-18 cells presented dilated vesicles only 50% of the PC-3E cells were affected. In addition, Golgi apparatus was still identifiable in the unaffected PC-3E cells. To further probe the status of the Golgi apparatus, we performed immunostaining of GM130, a marker of cis-Golgi cisternae, on cells treated with monensin (Fig. 4c). In absence of monensin, a similar punctate staining of GM130 located in the perinuclear region was observed in the two cell lines. After a 6 h exposure, monensin induced a dispersion of the signal in TEM 4-18 cells whereas the punctate staining was maintained in PC-3E cells even after 48 h (Fig. 4d). Taken together, these results suggested that an early effect of monensin in TEM 4-18 cells is associated with a disruption of Golgi apparatus, potentially related to an osmotic swelling of Golgi cisternae.

### Monensin does not induce ferroptosis

**Figure 5:**
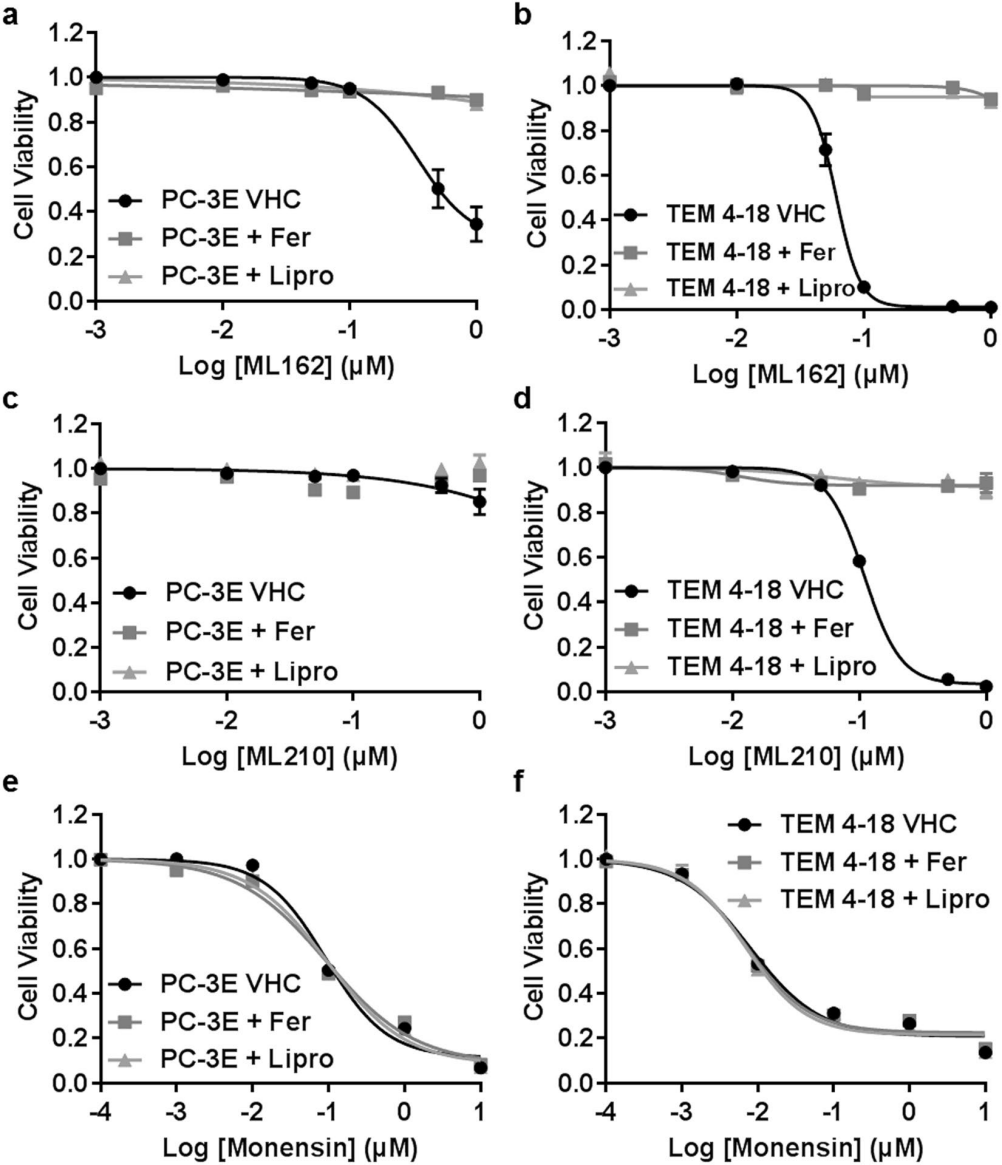
Monensin and GPX4 inhibitor EMT-selective toxicity involves different mechanisms. Cells were treated for 72 h with serial dilution of ML162 (**a**,**b**), ML210 (**c**,**d**) or monensin (**e**,**f**) alone or in combination with vehicle (VHC) ferrostatin 2 µM (Fer), or liproxstatin 0.5 µM (Lipro). Relative cell viability was plotted against the logarithm of drug concentration. The results represent the means ± SEM (n = 6).

With a similar objective but different approach, recent studies identified GPX4 inhibitors as selective targeting compounds against therapy-resistant high-mesenchymal cell state^16,17^. RSL3, ML210 and ML162 are compounds known to induce ferroptosis, a non-apoptotic, oxidative form of regulated cell death involving lipid hydroperoxides. The greater sensitivity of mesenchymal cells to such inhibitors suggests their dependency on a lipid-peroxidase pathway. Therefore, we wished to know if monensin also induced ferroptotic cell death. We tested the effect of two of the GPX4 inhibitors (ML162 and ML210) on our EMT-like and epithelial cells. Corroborating the aforementioned studies, TEM 4-18 cells were highly sensitive to both GPX4 inhibitors and this effect was totally reversed by inhibitors of ferroptosis (Ferrostatin-1 and Liproxstatin-1) (Fig. 5a–d). Conversely, the effects of monensin were not reversed by ferroptosis inhibitors suggesting that the cytotoxic effects of monensin do not stem from feroptosis and are not directly dependent on GPX4 activity (Fig. 5e,f).

### Monensin is an EMT-selective cytotoxic compound

**Figure 6:**
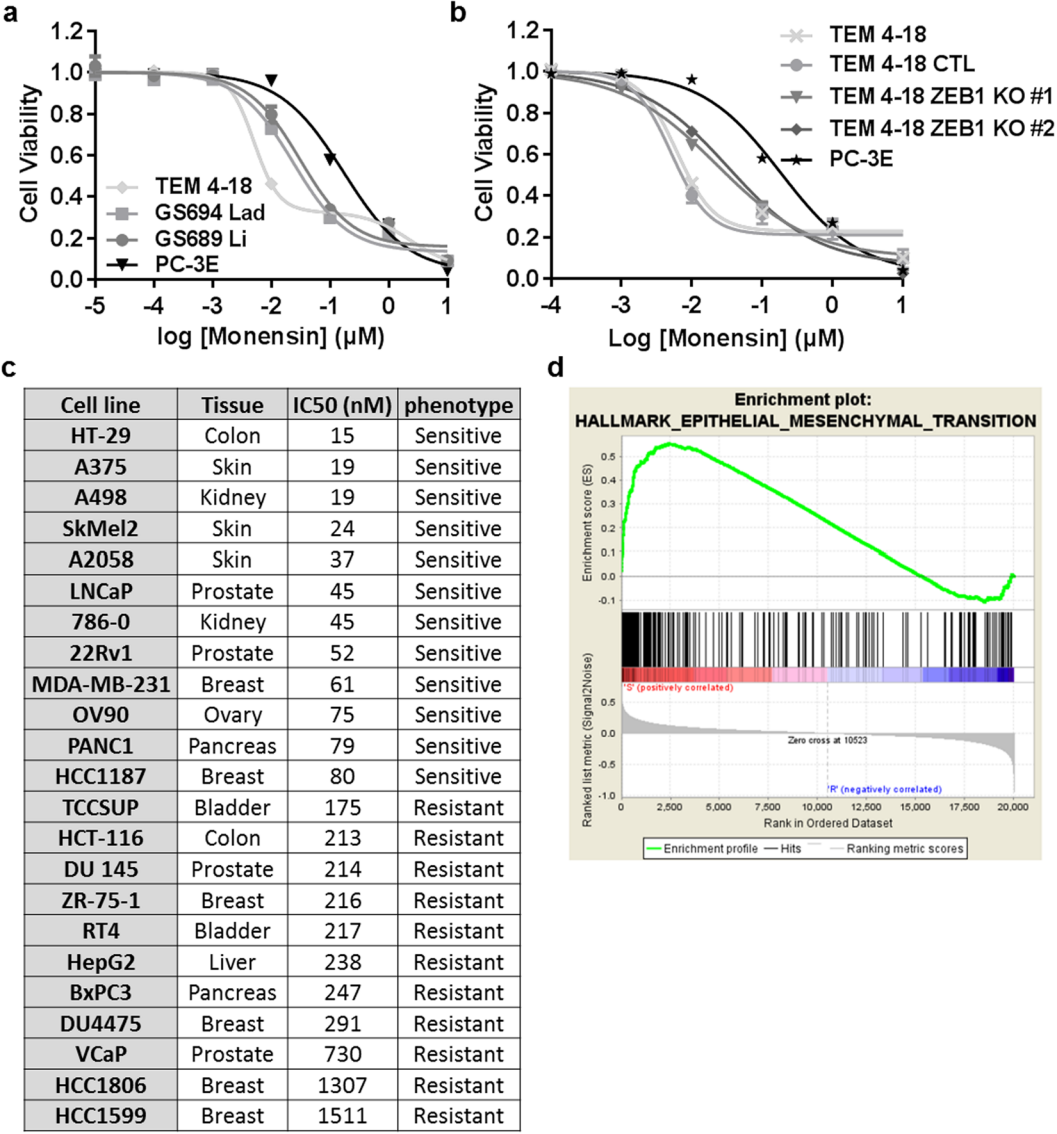
Monensin is selectively cytotoxic to a broad panel of cancer cell lines exhibiting EMT-like characteristics. (**a**,**b**) Cells were treated for 72 h with serial dilution of monensin (n ≥ 4). Relative cell viability was plotted against the logarithm of monensin concentration. (**c**) 23 human cancer cell lines were tested for their sensitivity to monensin (n ≥ 3). Cell lines were grouped as sensitive (IC_50_ < 100 nM) or resistant (IC_50_ > 100 nM). (**d**) The Cancer Cell Line Encyclopedia database was used to perform a Gene Set Enrichment Analysis. The hallmark for EMT was the top of the hallmark enriched in sensitive cell lines. The results represent the means ± SEM of at least 3 independent experiments.

In order to test the generality of our findings, we evaluated the toxicity of monensin across a wide range of cancer cell lines, representing a spectrum of epithelial to mesenchymal phenotypes. We first tested monensin on two cell lines (GS694 Lad, GS689 Li) established from metastatic tumors in mice after intravenous injection of the parental PC-3 cell line. Our results indicated that the two *in vivo* passaged cell lines, which display a high metastatic potential as well as an EMT-like phenotype^9^, exhibited a greater sensitivity to monensin compared to the epithelial PC-3E cells (Fig. 6a). In addition, genetic ablation of the EMT transcription factor ZEB1 in TEM 4-18 cells reversed the sensitivity of the cells to monensin (Fig. 6b). While ZEB1 KO significantly increased the IC_50_ to monensin in independent clones by (#1: 4.6 fold; #2: 6 fold) when compared to their respective control, TEM 4-18 ZEB1 KO cells still remained more sensitive to monensin compared to the epithelial PC-3E cells. As all the aforementioned cell lines were derived from the parental PC-3 cell line, we extended our study to a broader panel of human cancer cell lines. Dose-response experiments were performed to evaluate the toxicity of monensin in 23 cancer cell lines and, based on the calculated absolute IC_50_, the cell lines were grouped into a sensitive (IC_50_ < 100 nM) or resistant (IC_50_ > 100 nM) group (Fig. 6c). To explore whether monensin toxicity is correlated with cell growth rate (as a function of growth medium used (Supplementary Fig. S3) or otherwise), we evaluated the sensitivity of the cell lines to monensin by measuring the growth rate inhibition 50 (GR_50_), which accounts for differences in growth rates^18^. We note that across this panel of cell lines, monensin-sensitivity as measured by IC_50_ and GR_50_ were well correlated (Supplementary Fig. S3). We then analyzed publically-available gene expression data (Cancer Cell Line Encyclopedia) and performed a Gene Set Enrichment Analysis to identify the gene sets differentially represented in the 2 groups. Supporting our hypothesis that monensin is selectively toxic to cells in an EMT-like state, the gene set “Hallmark Epithelial Mesenchymal Transition” was the top gene set enriched in the sensitive group (Fig. 6d). To confirm the results of the gene set enrichment analysis, we evaluated the expression of the epithelial marker E-cadherin and the expression of two EMT markers, vimentin and ZEB1, at the protein level across the cell line panel (Supplementary Fig. S4). Our results confirmed that the cell lines classified as sensitive tend to share EMT characteristics such as a decreased expression of E-cadherin and increased expression of vimentin and ZEB1 (Supplementary Fig. S4). However, we observed some cell lines displaying epithelial characteristics, such as HT-29, LNCaP and 22Rv1 cells, that are sensitive to monensin. This suggests that the mechanisms leading to monensin sensitivity are not exclusive to an EMT-like phenotype or that these cells lines share some characteristics with EMT-like cells. On the other hand, all the cell lines tested that display EMT characteristics were in the sensitive group with the exception of DU145 cell line, which is known to be heterogeneous and possess cells with an EMT-like phenotype^19^. Moreover, selective disruption of the Golgi apparatus by monensin were observed in other EMT-like cell lines (Supplementary Fig. S5). Altogether, our results suggest that cell lines sensitive to monensin share hallmarks of EMT.

### Monensin sensitivity is correlated with increased cellular uptake in EMT-like cells

**Figure 7:**
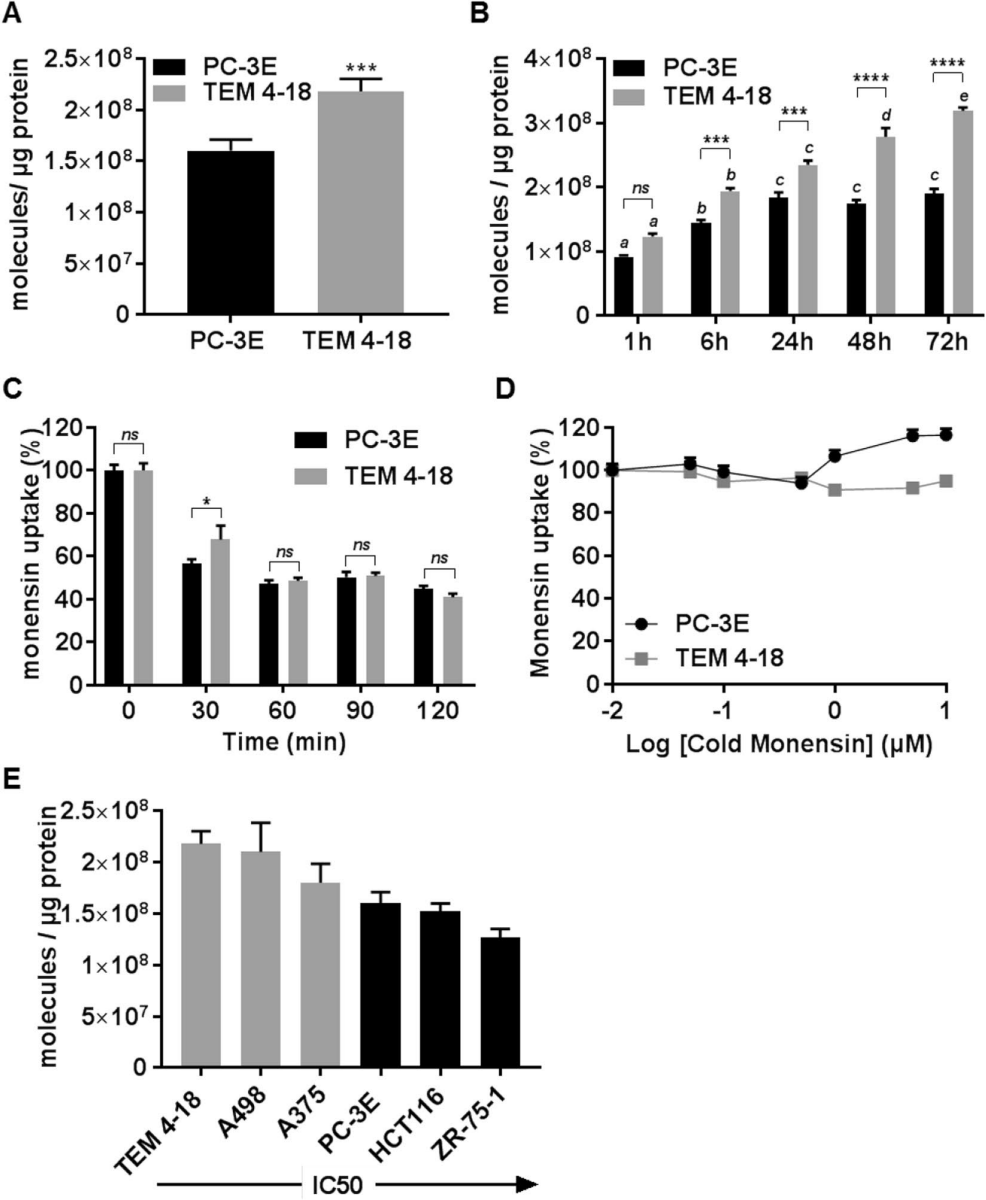
Monensin sensitivity is correlated with increased cellular uptake in EMT-like cells. Cellular uptake of ^3^H-monensin in cells treated with ^3^H-monensin (10 nM) for (**A**,**E**) 6 h or (**B**) 1 h to 72 h. (**C**) Cells were treated with ^3^H-monensin (10 nM) for 6 h. Cellular monensin retention was measured 0, 30, 60, 90 and 120 min after the media was replaced. (**D**) Cells were incubated 6 h with ^3^H-monensin (10 nM) in presence of increasing concentration of cold monensin. Results were expressed in percentage of the radioactivity measured in cells treated with ^3^H-monensin only. Data represent mean values ± SEM of at least 3 independent experiments. *p < 0.05, **p < 0.01, ***p < 0.005, ****p < 0.0001. Different letters on top of bars indicate significant differences among time points (*p* < 0.05) (n ≥ 3).

One of the possible mechanisms that could account for increased sensitivity to monensin in EMT-like cells is a difference in its cellular uptake. In order to test this hypothesis, we evaluated the cellular uptake of ^3^H-monensin in TEM 4-18 and PC-3E cells. As cell size differs between these cell types^20^, we normalized radiolabeled monensin uptake to protein content. The amount of monensin uptake after a 6 h exposure to 10 nM ^3^H-monensin uptake was about 1.4-fold greater in TEM 4-18 than in PC-3E cells (Fig. 7A). Interestingly, monensin uptake increased throughout a 72 h observation period in TEM 4-18 cells, whereas in PC-3E cells uptake plateaued at 24 h (Fig. 7B). Differential uptake at later time points was not related to a greater capacity of PC-3E cells to expel monensin via an active transport mechanism. Indeed, a retention assay indicated that the level of intracellular monensin dropped at the same rate in both TEM 4-18 and PC-3E cells, decreasing by half 60 min after ^3^H-monensin was removed from the incubation media (Fig. 7C). Although the chemical nature of monensin suggests that it enters passively into cell membranes, we performed a saturation binding assay to examine whether monensin uptake involves active transport mechanisms. We incubated the cells with 10 nM ^3^H-monensin in the presence of increasing concentration cold monensin (50 nM to 10 µM). Monensin uptake was not saturable, as the uptake of ^3^H-monensin was identical despite the presence of 1000-fold excess of non-labelled monensin (Fig. 7D). These results indicate that the differential uptake of monensin in EMT-like cells is not due to differential expression of a transporter. Finally, we studied monensin uptake in other sensitive (A375, A498) and resistant (ZR-75-1, HCT116) cell lines. As predicted by the results above, monensin uptake correlated positively with the sensitivity status of the cells, sensitive cells displaying the higher intracellular content of monensin compared to resistant cells (Fig. 7E). Altogether, these results suggested that a differential monensin uptake could contribute to the selective toxicity of the compound against EMT-like cells.

## Discussion

To identify potent and selective cytotoxic compounds for prostate cancer cells exhibiting an EMT-like phenotype, we developed a high content HTS using a coculture of epithelial and EMT-like cells previously isolated from the parental PC-3 prostate cancer cell line. We identified monensin and salinomycin, two monovalent cation ionophores, as potent inhibitors of EMT-like cells. We further evaluated nigericin, a closely related compound absent from the compound library, which appeared to be the most potent of the three compounds tested. These ionophores exhibit distinct monovalent ion preferences: monensin (10Na^+^ > K^+^), salinomycin (10 K^+^ > Na^+^), and nigericin (100 K^+^ > Na^+^)^21^. Although our study revealed that monensin and nigericin were more potent than salinomycin; it is not obvious from the ion selectivity profile that this property of these compounds can explain the differential potency. While these compounds have never been intentionally used in humans, they are widely used (especially monensin) in agriculture as an anticcocidosis agent in poultry and growth promoter in cattle^22^. In 2009, Gupta *et al*. identified salinomycin (8-fold selectivity), and to a lesser extent nigericin (2-fold selectivity), as selective cytotoxic compounds for EMT-like Cancer Stem Cells (CSCs) using HTS^23^. Prior to this study, the same laboratory made the initial connection between EMT-like and CSC behavior, proposing that EMT may be a mechanism by which cancer cells gain CSC traits^24^. Indeed, the HTS in Gupta *et al*. was performed using an EMT-like model as a surrogate for CSCs. Since 2009, salinomycin has been used as a positive control in several anti-EMT or anti-CSC screens^25–27^. To date, the anti-CSC activity of salinomycin has been associated with induction of cell death, DNA damage and cell cycle arrest, as well as with the inhibition of Hedgehog and Wnt signaling pathways in various types of cancers^28–31^. Monensin was identified as a selective antineoplastic agent in prostate cancer cell line models^32^ and more recently as a selective inhibitor of cancer stemness in induced cancer stem-like tumorspheres^27^. The anti-EMT or anti-CSC properties of nigericin have been studied in different models^33–36^. Thus, this family of monovalent cation ionophores has repeatedly demonstrated cytotoxic effects against cancer cells exhibiting EMT-like or CSC phenotypes although the precise mechanism(s) remain unclear.

Monensin is a metabolite of *Streptomyces cinnamonensis* that binds protons and monovalent cations such as Na+, K+. Monensin acts as an electroneutral exchange diffusion carrier, mediating one-for-one cation exchange. We showed that, at 10 nM, monensin induced a cell cycle arrest, apoptosis and formation of reactive oxygen species in the TEM 4-18 cells within 72 h without having any effect on their epithelial counterparts. A clonogenic assay further indicated that irreversible deleterious effects of monensin at this concentration appeared as early at 6 h. Such effects of monensin on growth inhibition, apoptosis, cell cycle and oxidative stress have previously been described in multiple cancer cell lines including the parental PC-3 cell line^11,37–39^. However, it is notable that these effects were typically evaluated at much higher doses of monensin compared to the 10 nM dose used in our study. One of the earliest and most dramatic subcellular effects of monensin we noted was a disruption of the Golgi apparatus in EMT-like cells characterized by the accumulation of swollen vesicles in the cytoplasm. The absence of intact Golgi cisternae as well as a disruption of the cis-Golgi marker GM130 localization supported the Golgi-derived nature of these vesicles. Vacuolation of Golgi apparatus cisternae is described in the literature as one of the first subcellular effects observed after exposure to monensin^15^. Monensin has long been known as a Golgi-disrupting agent that induces a Golgi stress response, perturbs the structure and function of the Golgi apparatus, and subsequently affects protein trafficking^14,15,40^. Monensin-induced swelling of Golgi membranes has been associated with alteration of intracellular transport of secretory proteins which accumulate in the Golgi complex^41^. In addition, posttranslational Golgi-associated processing events can also be affected following exposure to monensin due to the disruption of Golgi pH. In response to such cellular stress, the Golgi complex may initiate signaling pathways to trigger apoptosis^42^. Monensin has notably been shown to induce a Golgi stress response and genes involved in apoptosis induction were identified among the genes whose transcription was enhanced in response to disruption of Golgi function^40^. However the precise mechanisms leading to the massive swelling and disruption of the Golgi apparatus after monensin exposure are still unclear. It has been shown that a pH gradient is maintained along the secretory pathway^43^. This gradient is established by proton pumps and is highly regulated and important for Golgi apparatus function. The trans-cisternae of the Golgi apparatus and newly formed secretory vesicles have an acidic pH^44–46^. As an ionophore able to dissipate proton gradients, monensin can increase the pH of these acidic compartments. By exchanging Na^+^/H^+^ across membranes, monensin promotes Na+ influx along with water leading to swelling of the cisternae^47^. As monensin-induced Golgi membrane swelling is the consequence of the regulated maintenance of pH gradients along the secretory pathway, a differential expression or activity of pH sensors or regulators could also drive monensin sensitivity. For example, it has been shown that adriamycin-resistant tumor cells (MCF-7adr) derived from the MCF-7 line display a higher pH gradient between the vesicular luminal compartments and the cytoplasm compared to the parental MCF-7 cells^48^.

We also observed early effects of monensin on mitochondria—including an increase in mitochondrial membrane potential in sensitive TEM 4-18 cells and elevated expression of the mitochondrial damage marker BNIP3 in both resistant PC-3E and sensitive TEM 4-18 cells. Mitochondrial membrane potential reflects the electrochemical gradient that exists between the inner and outer membrane of mitochondria. This gradient is directly dependent on the pH gradient in normal conditions. However, monensin affects the pH gradient without affecting the electrochemical gradient, by exchanging protons for monovalent cations. This suggested that the electron transport chain increased its activity to restore the pH gradient resulting in an increase of the electrochemical gradient. Thus, an increased mitochondrial membrane potential could reflect a homeostatic response to monensin rather than event triggering cell death. However, both TEM 4-18 and PC-3E cells exhibit increased mitochondrial damage in <24 h exposure as revealed by BNIP3 immunoreactivity. This suggests that TEM 4-18 cells might be more sensitive to mitochondrial damage than PC-3E cells. Indeed, we observed that PC-3E cells exhibit more BNIP3 immunoreactivity without monensin exposure compared to TEM 4-18 cells. It is therefore difficult to determine whether the irreversible and selective toxicity of monensin is due to disruption of mitochondria, Golgi membranes or both pH-dependent organelles. Our studies indicate that disruption of another pH-dependent organellar event -fusion of autophagosomes with lysosomes-does not contribute to the loss of cell viability (data not shown).

Regardless of what cellular compartment(s) is/are crucially targeted by monensin, EMT-like cells appear to have a particular vulnerability to this compound. In part, this may stem from our novel observation that EMT-like cells exhibit greater uptake of monensin, and thus the intracellular concentration of this drug is higher in EMT-like cells. We showed that this elevated cellular uptake is not a function of active transport into or out of cells. Consistent with the notion that the lipophilic nature of monensin allows it to diffuse directly into cell membranes, monensin uptake was not saturable. Although the 1.4-fold greater cellular uptake may seem like a modest increase, this could reflect partitioning of monensin into specific membrane domains that lead to its deleterious effects. Some evidence indicates that monensin tends to be inserted into cholesterol-rich membranes^49^ and that cholesterol is accumulated in the Golgi apparatus^14,50^. Interestingly, swollen vesicles formed after monensin treatment were found to be remarkably rich in cholesterol^40^. Thus, differences in membrane lipid composition might target the effects of monensin to particular organelles. Emerging evidence indicates that EMT-like cells exhibit a variety of perturbations in lipid metabolism^51^. Cells in an EMT-like or mesenchymal state have been shown to be sensitive to statins^16,52,53^. Moreover, a recent study indicates that a common mechanism of drugs that can inhibit an EMT gene expression signature is disruption of membrane fluidity which depends on cholesterol content^26^. Interestingly, the lipid peroxidase GPX4, was identified as a key metabolic target for cells in a mesenchymal state^16^. GPX4 inhibitors selectively induce ferroptosis in various drug-resistant and mesenchymal cell states in a manner that depends on ZEB1^16^. Here we show that monensin sensitivity is also partially dependent on ZEB1, but monensin does not induce ferroptosis in our EMT-models like GPX4 inhibitors. This indicates that the mechanisms by which monensin and GPX4 inhibitors target EMT-like cells are related, but distinct. Interestingly, cell death induced by salinomycin has recently been shown to be partially prevented by the ferroptosis inhibitor ferrostatin-1 in breast CSCs, suggesting that monensin and salinomycin may have different mechanisms of action^54^. Our findings suggest the possibility that differences in lipid metabolism or compartmentalization may be a clue to understanding the differential sensitivity of monensin and other EMT-selective agents. This chemical biology approach reveals phenotypic features of cancer cells that might be exploited for therapeutic purposes.

## Methods

### Cell culture and reagents

PC-3, 22Rv1, LNCaP, VCaP, DU145, MDA-MB-231, ZR-75-1, BxPC3, 786-O, A498, HCT-116, HT-29, A2058, SkMel2, A375, OV90, PANC1, HCC1187, TCCSUP, RT4, HepG2, DU4475, HCC1806 and HCC1599 were obtained from the American Type Culture Collection (ATCC, Manassas, VA). Cell lines derived from PC-3 (PC-3E and TEM 4-18^9^; GS689Li and GS694Lad^9^; TEM 4-18 ZEB1 KO) were previously generated in our laboratory. The identity of PC-3E, TEM 4-18, GS689Li and GS689Lad as PC-3 derivatives wes confirmed by STR profiling (IDEX Laboratories). All cell lines were grown at 37 °C in a 5% CO_2_ atmosphere in ATCC-specified medium containing 10% fetal bovine serum and 1% non-essential amino acids. Monensin (M5273, 90–95% purity), salinomycin (S4526), nigericin (N7143), pyrvinium pamoate (1592001), epirubicin hydrochloride (E9406), gentian violet (48770), dactinomycin (1162400) and daunorubicin (D8809) were purchased from Sigma (Saint-Louis, MO).

### Cell line engineering

The ORF of eGFP and mCherry were obtained by PCR from pEGFPC1 and pRSETB, and subcloned into a retroviral expression vector pQCXIP (Clontech, Mountain View, CA). GP2-293 retrovirus packaging cells were cotransfected with retroviral vector pQCXIP-eGFP, pQCXIP-mCherry or pQCXIH empty vector and pVSV-g plasmid using Lipofectamine 2000 (Invitrogen, Carlsbad, CA) following the manufacturer’s instruction. PC-3E and TEM 4-18 cells were infected with eGFP and mCherry retrovirus respectively and selected with puromycin (1 µg/ml) to established stable cell lines. PC-3E GFP cells were further infected with pQCXIH retrovirus and selected with 250 μg/ml hygromycin to make cells hygromycin resistant.

ZEB1 KO TEM 4-18 cells were engineered using the CRISPR/Cas9 system. Cells were transfected with 1.0 µg of The ZEB1 CRISPR/Cas9 KO plasmid and ZEB1 HDR plasmid (Santa Cruz Biotechnology, Santa Cruz CA) using PolyFect transfection reagent (Qiagen, Hilden Germany). After 72 h, transfected cells were selected with 1.0 µg/ml puromycin. Loss of ZEB1 expression in puromycin resistant clones was confirmed by Western blotting and sequencing of the ZEB1 locus. TEM 4-18 control cells were engineered by cotransfecting cells with 1.0 µg of the control plasmid and ZEB1 HDR plasmid (Santa Cruz Biotechnology, Santa Cruz CA).

### Pilot High Througuput Screening (HTS), hit selection and hit confirmation

Mixed PC-3E GFP and TEM 4-18 mCherry cells (1:1) were seeded at 4000 cells/well in 96-well plates using Multiflo dispenser (Biotek, Winooski, VT). After 24 hours, cells were treated with the Microsource Spectrum compound collection (Microsource, Gaylordsville, CT) and UI Legacy compound collection (1 µM) using Microlab Star liquid handling robot (Hamilton Robotics, Reno, NV). Hygromycin in different dose (2 to 500 µg/ml) was used as a positive control. The plates were imaged on Operetta high-content wide-field fluorescence imaging system (Perkin Elmer, Waltham, MA) after 72 h routine incubation. For each well, 13 different fields were imaged with two channels (GFP and mCherry) using a 20x objective lens for epifluorescence imaging. Images were analyzed with the accompanying Harmony software (version 3.5) using custom made algorithms to determine numbers of PC-3E GFP and TEM 4-18 mCherry cells per field. Data across the screen were normalized to the average cell number of untreated control wells to get relative cell viability, and visiualized with TIBCO Spotfire (Perkin Elmer, Waltham, MA).

The robustness of the assay was validated by performing a Z’-factor analysis. TEM 4-18 mCherry cells were treated for 72 h with 350 µg/ml hygromycin or vehicle. Normalized cell number from both vehicle- and hygromycin-treated wells were used for Z-factor, signal-to-noise (S/N) and signal-to-background (S/B) ratio calculation (with S/N = (µ_n_ − µ_p_)/SD_n_, S/B = µ_n_/µ_p_, and Z’ factor = 1 − 3 * (SD_p_ + SD_n_)/(µ_n_ − µ_p_), in which SD_p_ and SD_n_ are standard deviations, and µ_n_ and µ_p_ are means of the negative and positive control wells). Reproducibility of the assay was further demonstrated by comparing the results of two screenings performed with an identical set of 80 compounds, and the dose-responses of the hygromycin in each plate.

Dose response experiments were performed exactly the same as in the pilot screen to validate available hits. Cells were treated for 72 h with serial dilution of compounds and resulted normalized cell number was plotted against tested drug concentration to obtain IC_50_ values, using GraphPad Prism 6 (La Jolla, CA).

### Cell Viability assay

PC-3E and TEM 4-18 cells were plated at 2000 cells/well in 96-well plates. After 24 h, cells were treated with a serial dilution series of monensin, salinomycin, nigericin, ML162 and ML210. Co-treatment with ferrostatin (2 µM) and liproxstatin (0.5 µM) were also performed. After 72 h, cell viability was determined using Cell-Titer-Blue assay (Promega) according to manufacturer’s instructions. The Synergy HT plate reader (Biotek, Winooski, VT) was used for signal quantification and the fluorescence values were normalized to the vehicle well for each cell line. Absolute IC_50_ were calculated with GraphPadPrism software (GraphPad Software, Inc.).

### Cell growth, cell cycle and apoptosis assay

PC-3E and TEM 4-18 cells were seeded at 50,000 cells/well in 6-well plates and left to attach overnight. Cells were then treated with 10 nM monensin for 24 h, 48 h and 72 h cells. At each time point, cells were harvested and cell concentration was calculated to obtain the number of cells/well. The population doubling level was calculated using the formula: PDL_n_ = 3.32 (log X_t_ − log X_0_) + PDL_n−1_ (with X_t_ = cell number at that time point, X_0_ = cell number used as inoculum and PDL_n−1_ = population doubling level at the previous passage). For cell cycle analysis, cells were fixed in ethanol 70%, treated with Rnase A, mixed with propidium iodide and processed with the Becton Dickinson LSR II flow cytometer. Analysis was performed with ModFit LT software. Apoptosis was assessed with the Annexin V Apoptosis Detection kit (sc-4252 AK, Santa Cruz Biotechnology, Dallas, TX) according to manufacturer’s instructions. Samples were processed with the Becton Dickinson LSR II flow cytometer and the analysis was performed with FlowJo software. Annexin V positive/Hoechst negative cells were counted as apoptotic cells.

### Measurement of the intracellular ROS levels

Intracellular ROS levels were measured using the fluoroprobe dihydroethidium (DHE) (Molecular Probes Inc., Eugene OR). PC-3E and TEM 4-18 cells were treated with 10 nM monensin for 72 h. At the end of the incubation, cells were harvested, and treated with PBS containing 5 mM pyruvate and 10 µM DHE probe for 40 min. Samples were processed with the Becton Dickinson LSR II flow cytometer. Analysis was performed with FlowJo software. Fluorescence values were normalized to the PC-3E vehicle.

### Mitochondrial membrane potential

A cytofluorimetric technic, using the lipophilic cationic probe 5,5′,6,6′-tetrachloro-1,1′,3,3′-tetraethylbenzimidazolcarbocyanine iodide (JC-1), was used to measure the mitochondrial membrane potential^55^. PC-3E and TEM 4-18 cells were treated with 10 nM monensin for 6 h, 24 h, 48 h and 72 h. At the end of the incubation, cells were incubated with 3 μM JC-1 probe (Molecular Probes Inc., Eugene OR) for 15 min at 37 °C. Cells were then harvested, resuspended in PBS and processed with the Becton Dickinson LSR II flow cytometer. Analysis was performed with FlowJo software. Fluorescence values were normalized to the PC-3E vehicle.

### Clonogenic assay

PC-3E and TEM 4-18 cells were treated with 10 nM monensin for 6 h, 12 h, 24 h, 48 h and 72 h. At the end of the incubation, the surviving cells were harvested and re-seeded in 6-well plates at 200 cells/well. After 11 to 13 days, cells were fixed with ethanol 70% and stained with Coomassie Brillant Blue R-250 staining solution (Bio-Rad, Hercules, CA). Colonies, defined by group of more than 50 cells, were counted for each well and normalized to untreated wells.

### Western Blot

Protein lysates were by scrape harvesting in lysis buffer. 20 to 50 µg of protein were separated on SDS-polyacrylamide gels (NuPAGE^®^ 4-12% Bis-Tris Protein Gels, Novex) and transferred on PVDF membranes (Immobilon-FL) prior to incubation with primary antibody overnight at 4 °C (Table 1). Membranes were then incubated with corresponding secondary antibody for 1 h at room temperature. The membranes were scanned with an Odyssey infrared imaging system (LI-COR Biosciences, Lincoln NE) and expression of protein was normalized to actin expression.

**Table 1:** List of antibodies used for this study.

Target	Source	Dilution
Β-Actin	clone AC-15, A1978, Sigma (Saint-Louis, MO)	1/10000
PARP	#9542, Cell Signaling Technology (Danvers, MA)	1/1000
GM130	Clone 35/GM130, #610822, BD Transduction Laboratories	1/1000
BNIP3	clone 670621, MAB4147, R&D systems (Minneapolis, MN)	1/1000
ZEB1	NBP1-88845, Novus (Saint Charles, MO)	1/1000
E-Cadherin	Clone 180215, MAB1838, R&D systems (Minneapolis, MN)	1/1000
Vimentin	AMF-17b, Developmental Studies Hybridoma Bank (Iowa City, IA)	1/500

### GM130 Immunofluorescence

Cells were seeded on glass coverslips and treated with 10 nM monensin for 1 h, 6 h, 24 h or 48 h. At the end of the incubation, cells were fixed in 4% PFA, permeabilized in PBS-0.2% Triton X100, and blocked in PBS-2.5% BSA prior to antibody incubation. Cells were incubated with a mouse anti-GM130 antibody (Table 1) and then with a donkey anti-mouse Cy3 antibody (Jackson Immuno Research, 715-166-150) at 1/500 in PBS-2.5% BSA for 1 h at room temperature. The coverslips were then mounted on cover glass with a DAPI (4′,6-diamidino-2-phenylindole)-containing mounting media and imaged with a Zeiss LSM710 confocal microscope. To quantify the signal, images were converted to a binary images and number of particles was determined using ImageJ.

### Transmission Electron Microscopy

PC-3E and TEM 4-18 cells were treated with 10 nM monensin for 6 h and 48 h. At the end of the incubation cells were prefixed with glutaradehyde 2.5% in sodium cacodylate 0.1 M overnight at 4 °C, post-fixed with 1% osmium tetroxide and 1.5% potassium ferrocyanide for 30 min and negatively stained with uranyl acetate 2.5% for 5 min. After dehydration with 25–100% alcohol, the cells were embedded in pure epon 812 resin. Once polymerized, the samples were sectioned, mounted on grids, stained with uranyl acetate 5% and lead citrate, and then observed under a transmission electron microscope (JEOL JEM-1230).

### Monensin upake

Cells were treated with 10 nM tritiated monensin ([^3^H]Monensin, 3 Ci/mmol) (American Radiolabeled Chemicals, Saint Louis, MO) for 1–72 h. At the end of the incubation, cells were washed twice with cold PBS 5% BSA and lysed with 0.5 M NaOH. Cell lysate was mixed with econo-safe™ counting cocktail (Research Product International, Mount Prospect, IL) and radioactivity was counted for 10 min with Tri-Carb 2800TR Liquid Scintillation Analyzer (PerkinElmer, Waltham, MA). Control plates, treated with non-radioactive monensin, were used in parallel to determine the amount of protein per plate. Results were expressed in molecules of monensin per µg of protein. For the retention experiment, cells were incubated 6 h with 10 nM [^3^H]Monensin. Cell lysate was then harvested 0, 30, 60, 90 and 120 min after the media was replaced. Data were expressed as percent of [^3^H]-monensin at 0 min. For the [^3^H]-monensin uptake competition experiment, cells were incubated 6 h with 10 nM [^3^H]-monensin in presence of increasing concentration of non-radioactive monensin (50 nM to 10 µM). Samples were processed as described above and results were expressed as percent of [^3^H]-monensin uptake in cells treated with [^3^H]-monensin only.

### Gene Set Enrichment Analysis

23 cell lines tested in our laboratory for sensitivity to monensin were grouped into sensitive (N = 13) or resistant (N = 11) group. Gene expression datasets for these cell lines were downloaded from the Cancer Cell Line Encyclopedia (CCLE) and were subjected to Gene Set Enrichment Analysis (GSEA)^56^. The hallmark gene sets database (h.all.v5.0) from the Molecular Signatures Database (MsigDB) was used for enrichment analysis. The gene sets showing FDR of 0.25, a well-established cutoff for the identification of biologically relevant gene, were considered enriched between classes under comparison.

### Statistical Analysis

Statistical analysis was conducted using two-way ANOVA followed by Bonferroni test or an unpaired t test by GraphPad Prism (Graphpad, La Jolla, CA, USA). A *p* value < 0.05 was considered significant (*p < 0.05, **p < 0.01, ***p < 0.001, ****p < 0.0001).

## Supplementary information

supplementary information

## Data Availability

The primary HTS data were deposited in PubChem. (https://pubchem.ncbi.nlm.nih.gov/bioassay/1345086). The datasets analyzed in the current study are available at the Cancer Cell Line Encyclopedia (https://portals.broadinstitute.org/ccle).
