# Supplementary material for: High content screening identifies monensin as an EMT-selective cytotoxic compound
Source: Sci Rep. 2019 Feb 4;9:1200. doi: 10.1038/s41598-018-38019-y (PMC6361972; doi:10.1038/s41598-018-38019-y)
Supplement: Supplementary file 1 — supplementary information [file 41598_2018_38019_MOESM1_ESM.pdf]

High content screening identifies monensin as an EMT-selective cytotoxic compound

Marion Vanneste, Qin Huang, Mengshi Li, Devon Moose, Lei Zhao, Mark A. Stamnes, Michael Schultz, Meng Wu, and Michael D. Henry

List of the supplementary material :

Supplementary Figure S1 : Optimization of high content imaging-based HTS.
Supplementary Figure S2 : Validation of HTS results.
Supplementary Figure S3 : Monensin IC50 is not correlated with growth rate of cells.
Supplementary Figure S4 : Expression of epithelial and mesenchymal markers in the resistant and sensitive cell lines.
Supplementary Figure S5 : Monensin sensitivity is correlated with alterations of the Golgi apparatus.
Supplementary Figure S6 : Unprocessed original scans used to generate Fig.3.
Supplementary Figure S7 : Unprocessed original scans used to generate Fig S4.

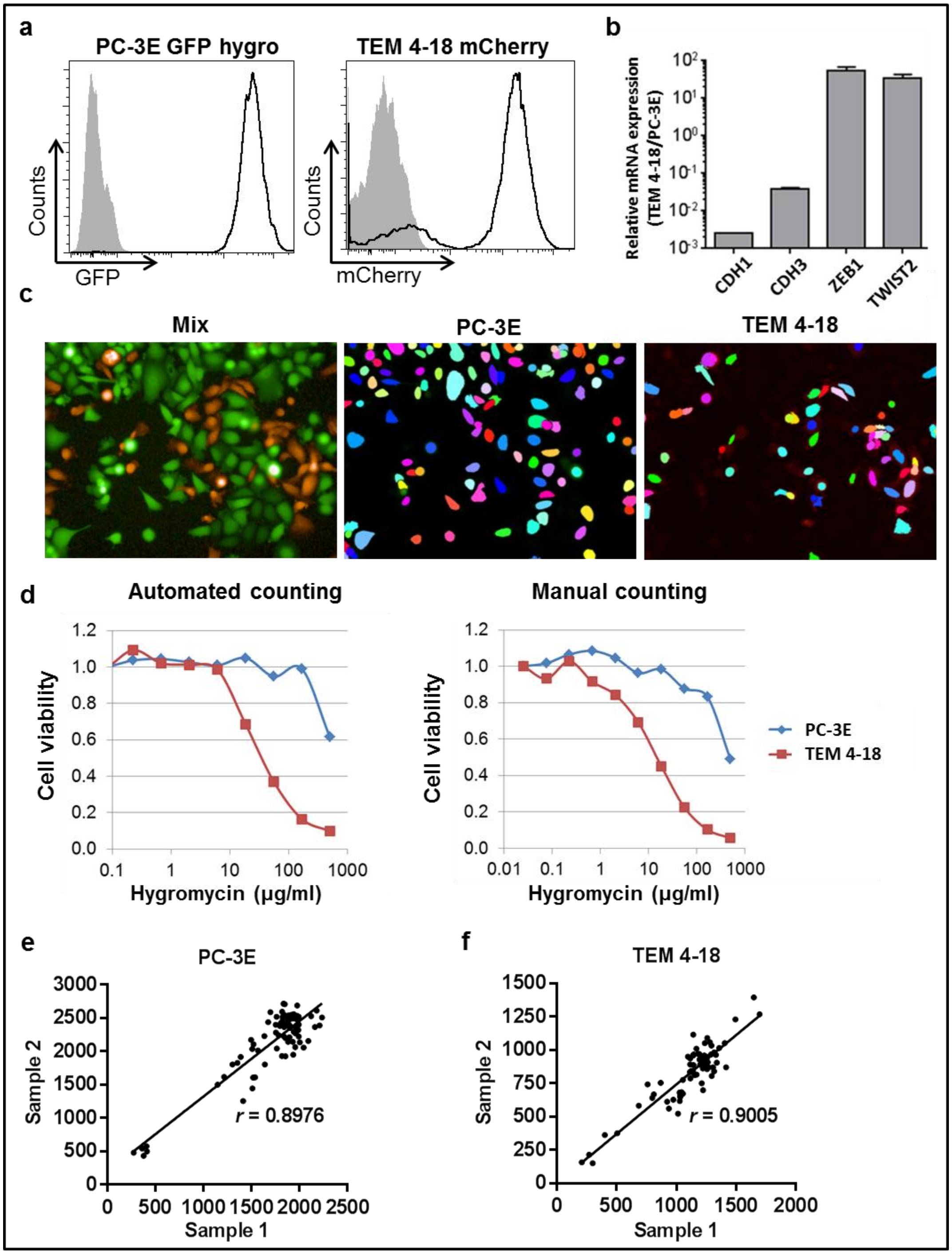

Supplementary Figure S1 : Optimization of high content imaging-based HTS. (a) Flow cytometry analysis validating expression of GFP and mCherry in PC-3E and TEM 4-18 cells respectively. (b) mRNA expression level of epithelial (CDH1, CDH3) and EMT (ZEB1, TWIST2) markers. Results are expressed as the ratio TEM 4-18 expression /PC-3E expression. (c) Identification of PC-3E GFP cells and TEM 4-18 mCherry cells by Perkin Elmer Harmony 3.5 software. (d) Comparison of automated counting (left panel) and manual counting (right panel) (n=1). Reproducibility of the assay was evaluated by comparing the results of two independent screens performed with an identical set of 80 compounds for both PC-3E (e) and TEM 4-18 (f) cells.

a

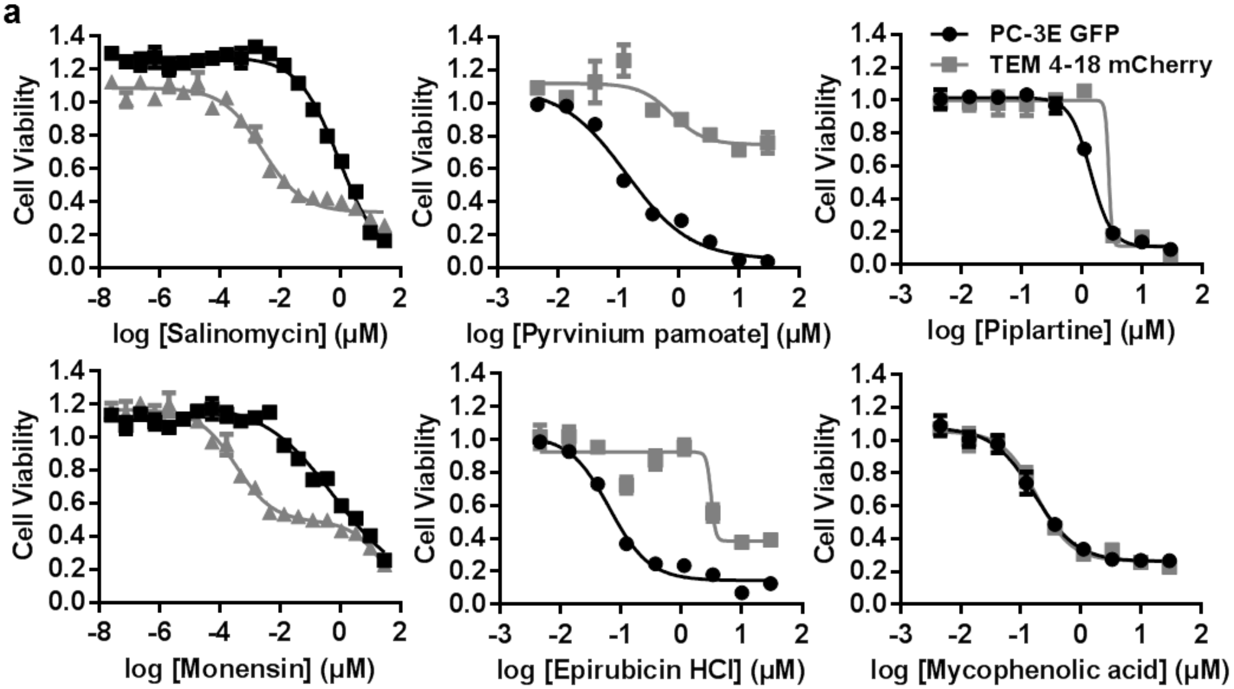

b

IC50 (nM)	PC-3E	TEM 4-18
Salinomycin	2028	18
Monensin	2636	76
Pyrvinium pamoate	190	> 10000
Epirubicin HCl	86	3483
Piplartine	1581	2877
Mycophenolic acid	344	327

Supplementary Figure S2 : Validation of HTS results. (a) Cells were treated for 72 h with serial dilution of compounds and relative cell viability was plotted against the logarithm of drug concentration. (b) Absolute IC_{50} values were determined for each compounds using GraphPad Prism 6. Data represent mean values ± SEM (n=3).

a

	PDT (h)	Growth media
**PC-3E**	24.1	DMEM/F12
**TEM 4-18**	28.1	DMEM/F12
**HT29**	24.9	DMEM
**HCT116**	16.3	DMEM
**22Rv1**	31.9	RPMI-1640
**A375**	18.3	DMEM
**A498**	34.5	DMEM
**ZR-75-1**	55.1	RPMI-1640
**TCCSUP**	37.0	RPMI-1640
**DU145**	27.1	RPMI-1640
**786-0**	22.0	RPMI-1640
**A2058**	26.3	DMEM
**BxPC3**	30.3	RPMI-1640
**SKMEL2**	61.5	DMEM
**LNCAP**	24.7	RPMI-1640
**MDA-MB-231**	27.8	RPMI-1640
**RT4**	31.5	McCoy's 5A
**HEPG2**	26.1	DMEM
**HCC1806**	27.0	RPMI-1640
**DU4475**	46.8	RPMI-1640
**HCC1187**	49.2	RPMI-1640
**PANC1**	36.3	DMEM

b

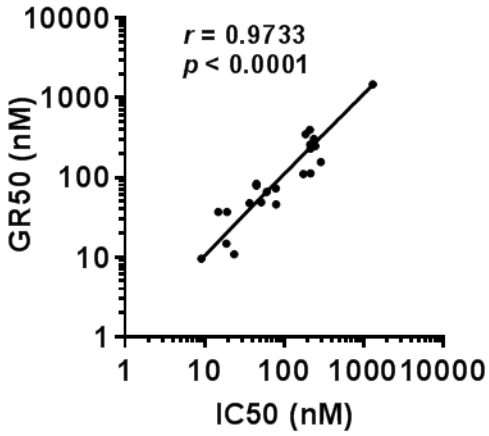

Supplementary Figure S3 : Monensin IC_{50} is not correlated with growth rate of cells. (a) The population doubling time (PDT) was calculated for most of the cell lines used for the GSEA. (b) Correlation between growth rate inhibition 50 (GR_{50}) and absolute inhibitory concentration 50 (IC_{50}). Data represent mean values ($n \geq 3$).

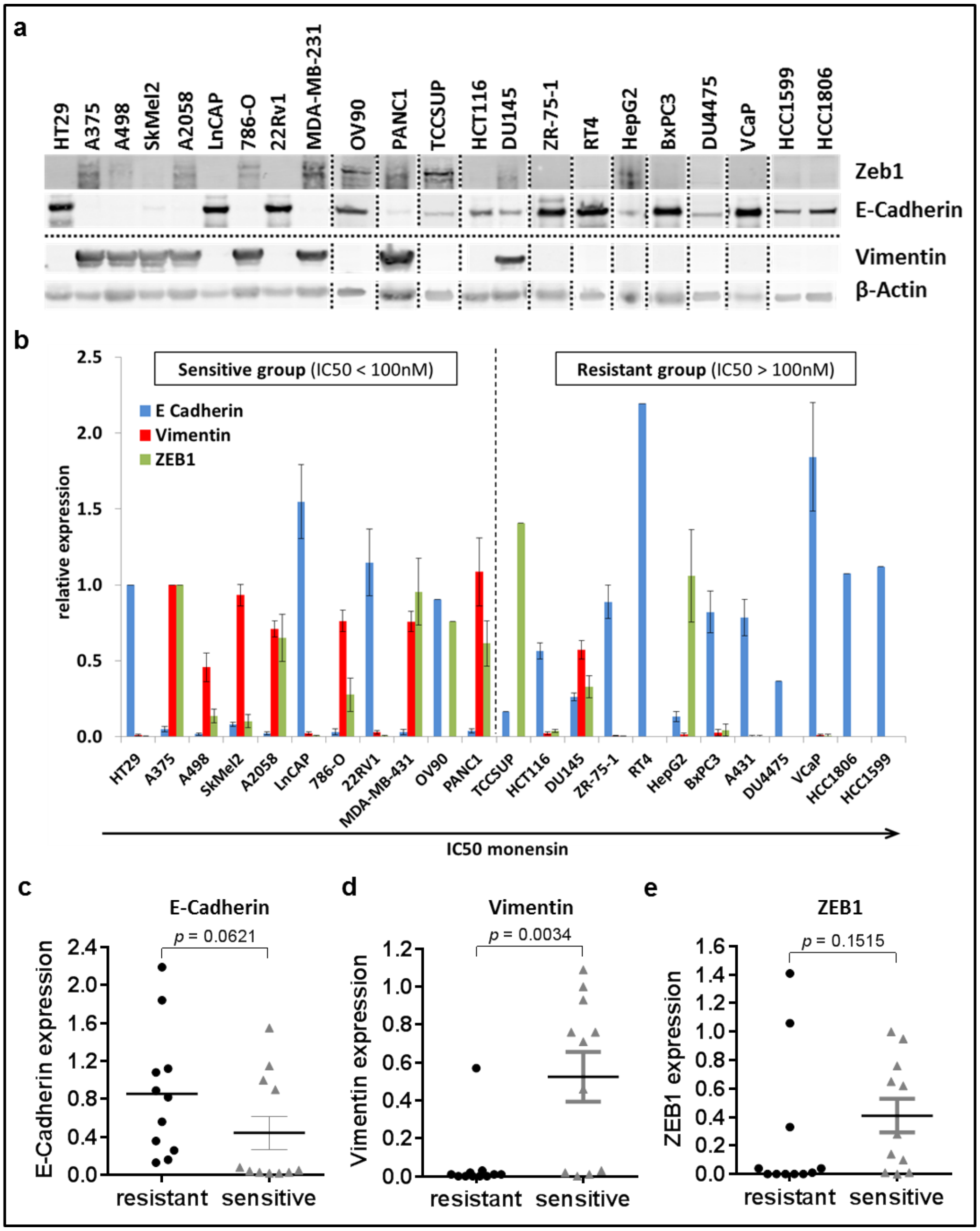

Supplementary Figure S4 : Expression of epithelial and mesenchymal markers in the resistant and sensitive cell lines. The level of Zeb1, E-Cadherin and Vimentin protein was assessed by Western blot. (a) shows a representative Western blot. The images shown are cropped from several blots (see Supplementary Fig. S7) as not all samples could be loaded on one gel, and the sections were rearranged by sensitivity to monensin. Also the blots were cut based on migration of molecular weight markers and stained separately (Zeb1/ E-Cadherin on one section ; Vimentin/ β-actin on a second section). (b) Protein expression was normalized to the expression of β-actin and expressed relatively to their level in HT29 (E-Cadherin) or A375 (Zeb1 and Vimentin) cells. On each blot, HT29 and A375 samples were loaded allowing comparison of the different cell lines across the multiples blots. Relative expression (y-axis) was plotted against sensitivity to monensin (x-axis). Data represent mean values ± SEM ($1 \leq n \leq 6$). (c-e) The mean expression of E-Cadherin (c), Vimentin (d) and Zeb1 (e) was compared between sensitive and resistant group (Mann-Whitney test).

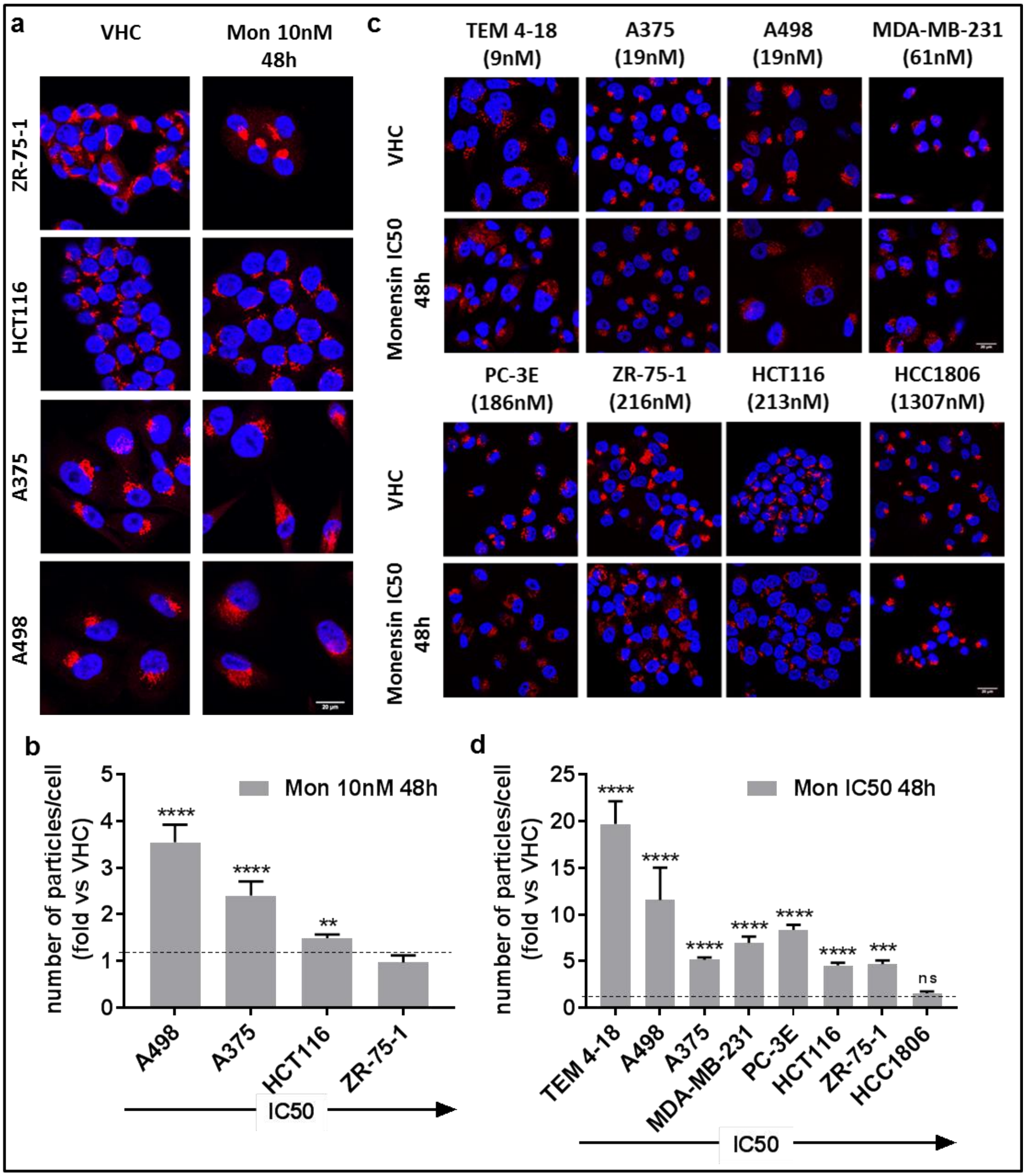

Supplementary Figure S5 : Monensin sensitivity is correlated with alterations of the Golgi apparatus. (a,b) Two resistant (ZR-75-1 and HCT116) and two sensitive (A375 and A498) cell lines were treated with monensin (10 nM) for 48 h. (a) Cells were stained for GM130 (red) and nucleus (blue). (b) GM130 signal was converted into binary signal and number of particles were counted using imageJ and normalized to the number of particles in untreated cells (n≥12). (c,d) Four resistant (PC-3E, ZR-75-1, HCT116 and HCC1806) and four sensitive (TEM 4-18, A375, A498 and MDA-MB-231) cell lines were treated with monensin at their respective IC_{50} for 48 h. (c) Cells were stained for GM130 (red) and nucleus (blue). (d) GM130 signal was converted into binary signal and number of particles were counted using imageJ and normalized to the number of particles in untreated cells (n≥33). Data represent mean values ± SEM. Scale bar: 20 μm. *p < 0.05, **p < 0.01, ***p < 0.005, ****p < 0.0001.

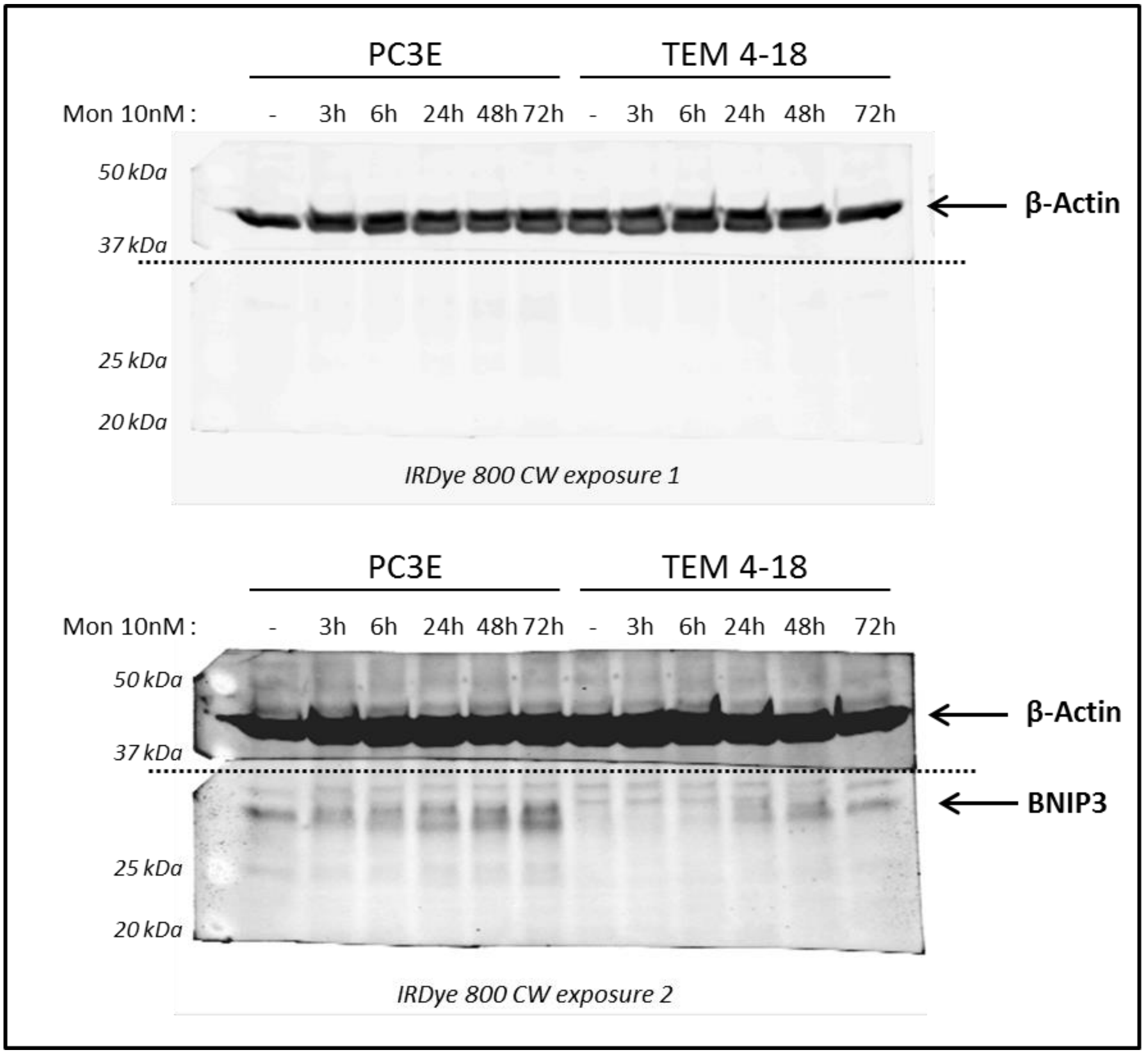

Supplementary Figure S6 : Unprocessed original scans used to generate Fig.3. Dash lines indicate where the membrane was cut to do the incubation with either β-Actin or BNIP3 antibody.

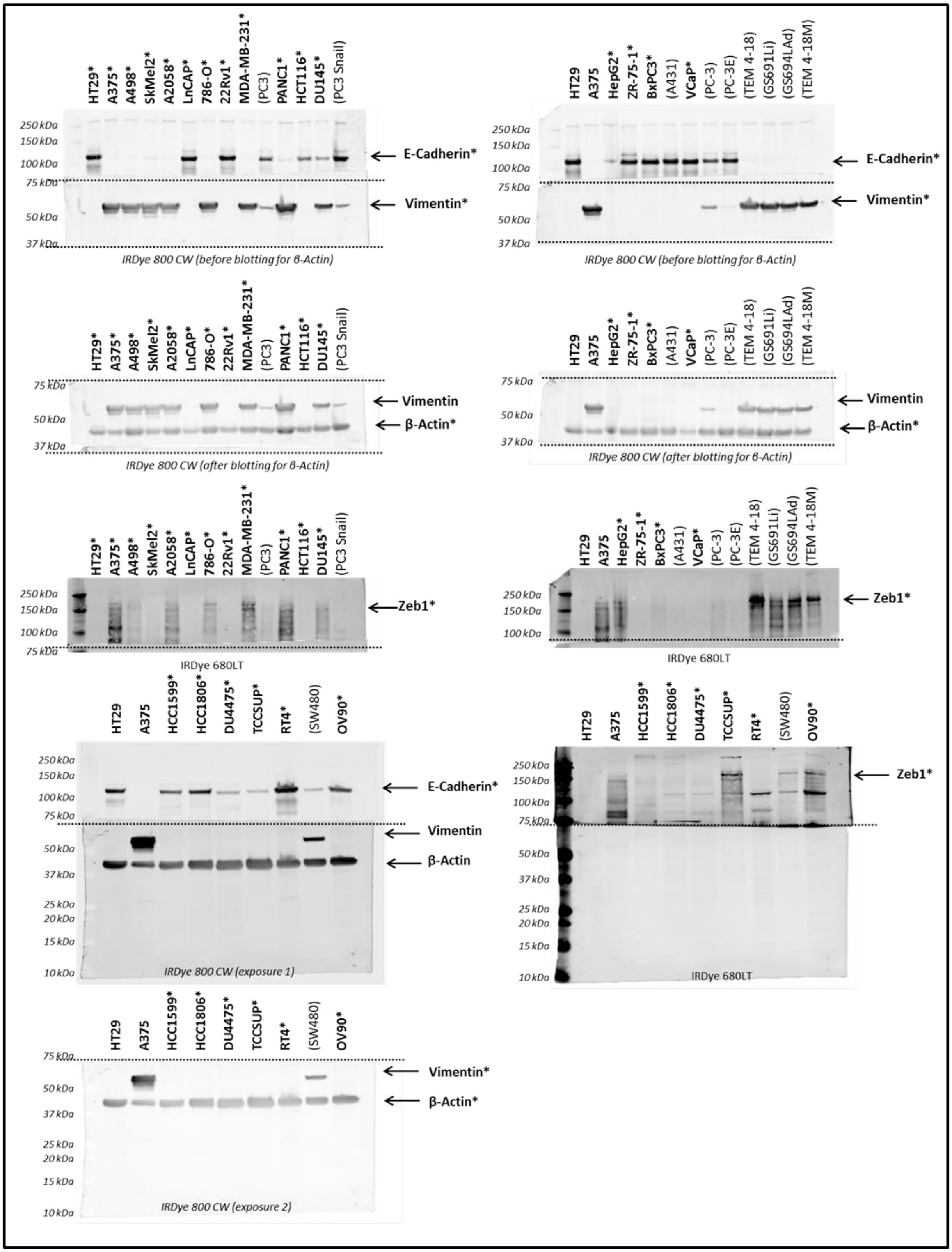

Supplementary Figure S7 : Unprocessed original scans used to generate Supplementary Fig. S4.
* denotes samples used to generate Supplementary Fig. S4 . Cell lines in parenthesis were evaluated but not part of Supplementary Fig. S4 (e.g. absent from the CCLE database). Dash lines indicate where membranes were cut to do the incubation with the different antibodies.
